# Drug-Induced Idiosyncratic Agranulocytosis - Infrequent but Dangerous

**DOI:** 10.3389/fphar.2021.727717

**Published:** 2021-08-13

**Authors:** Bernd Rattay, Ralf A. Benndorf

**Affiliations:** Department of Clinical Pharmacy and Pharmacotherapy, Institute of Pharmacy, Martin-Luther-University Halle-Wittenberg, Halle (Saale), Germany

**Keywords:** idiosyncratic agranulocytosis, adverse drug reaction, drug therapy safety management, agranulocytosis triggering drugs, agranulocytosis incidence

## Abstract

Drug-induced agranulocytosis is a life-threatening side effect that usually manifests as a severe form of neutropenia associated with fever or signs of sepsis. It can occur as a problem in the context of therapy with a wide variety of drug classes. Numerous drugs are capable of triggering the rare idiosyncratic form of agranulocytosis, which, unlike agranulocytosis induced by cytotoxic drugs in cancer chemotherapy, is characterised by “bizzare” type B or hypersensitivity reactions, poor predictability and a mainly low incidence. The idiosyncratic reactions are thought to be initiated by chemically reactive drugs or reactive metabolites that react with proteins and may subsequently elicit an immune response, particularly directed against neutrophils and their precursors. Cells or organs that exhibit specific metabolic and biotransformation activity are therefore frequently affected. In this review, we provide an update on the understanding of drug-induced idiosyncratic agranulocytosis. Using important triggering drugs as examples, we will summarise and discuss the chemical, the biotransformation-related, the mechanistic and the therapeutic basis of this clinically relevant and undesirable side effect.

## Introduction

Blood count changes often occur as a result of adverse drug reactions (ADRs). Although they are less frequent than other ADRs, their occurrence affects elementary physiological processes (blood gas transport, immune defense) and can thus lead to serious and severe adverse health effects. The most common blood count change observed as a side effect of drug use is a decrease in leukocyte counts (leukopenia). An epidemiological investigation showed that severe neutropenia or agranulocytosis associated with the use of drugs that are not part of the treatment in chemotherapy regimens occurs with mean incidence rates of 7.2 cases (2.3–15.4 cases) per million per year (Strom et al., 1992). In this context, the neutrophil granulocytes (neutrophils) are particularly affected. They represent the largest leukocyte fraction (approx. 50–70% of all leukocytes in healthy adults; approx. 2000–7,700/µL blood) and have a significantly shorter life span than lymphocytes. Therefore, leukopenia usually manifests as neutropenia, a deficiency of neutrophils in the blood. Neutropenia is present when the number of neutrophils in the blood falls below 1,500 cells/µL of blood. In individuals with black African ancestry, the average neutrophil counts are somewhat lower, so that values below 1,500 cells/µL of blood under specific circumstances can still be considered normal. This is referred to as benign ethnic neutropenia (BEN) (Reich et al., 2009). It was recently pointed out that a “BEN”-labelling might be problematic since it is sometimes regarded as a condition. This may lead to an inadequate treatment of non-caucasian patients in cases where drugs with neutrophil-lowering side effects (e.g., anti-cancer agents, clozapine) are usually indicated (Andreou, 2021; Merz and Achebe, 2021). We would therefore like to re-emphasize in this context that BEN represents a physiological feature.

As early as 1922, the physician Werner Schultz reported in the Berlin Association for Internal Medicine on several cases of gangrenous processes in the area of the oral and pharyngeal cavity with a high-grade reduction in the total number of white blood cells, almost exclusively affecting the granulocytes, and a fatal outcome after a short febrile illness (Schultz, 1922). He shaped the name agranulocytosis for the disease. Since then, numerous publications on the topic have appeared in the world literature. In this regard, agranulocytosis shows typical clinical features that can be used to diagnose the condition. It usually manifests as a severe form of neutropenia with neutrophil counts <500/µL blood. However, hematologists often only consider neutrophil counts of <100/µL blood (<0.1 × 10^9^/L) in association with fever or signs of sepsis to be “true” agranulocytosis (Andrès and Maloisel, 2008). The latter conditions are more commonly found in hospitalized patients. Agranulocytosis is essentially life-threatening. There is a high risk of severe infections as well as the risk of sepsis with septic shock. In rare cases, aplastic anemia with pancytopenia, a lowering of the cell count of all blood cells, may occur. The general risk of infection is already increased even with less pronounced reduction or deficiency of neutrophils. Usually, agranulocytosis is asymptomatic until the onset of infection. Fatigue, fever, chills, and muscle and joint pain are then often initial symptoms of the condition. These are often not taken seriously at first, since these symptoms are usually thought of as a flu-like infection. The typical symptom triad in agranulocytosis is characterized by 1) fever, which is often the only symptom, 2) sore throat and associated difficulty swallowing and 3) inflammatory mucosal lesions, e.g., aphthae (Stamer et al., 2017). It is not uncommon for neutrophil counts to increase before agranulocytosis occurs. This may be explained by the fact that a triggering infection induces the release of granulocytes that are still stored in the bone marrow. The earlier agranulocytosis is recognised, the more favourable is its prognosis. Cases with a fatal course are usually recognised late and consequently not treated optimally at an early stage.

## Main Triggers of Agranulocytosis—Dose-Dependent or Idiosyncratic Drug Reactions

In most cases, drugs are the main trigger of neutropenia or agranulocytosis. The results of studies that investigated the extent to which drugs induce neutropenia with a neutrophil count of <500/µL vary in the literature between 70 and 97% (Andersohn et al., 2004; 2007; Andrès and Maloisel, 2008; Curtis, 2014; 2017). These findings are important insofar as, when prescribing drugs with a known risk of agranulocytosis, a manifestation of agranulocytosis should always be considered when cold symptoms, especially fever, occur during therapy. Patients receiving such medicinal products should be instructed to contact a physician immediately if febrile symptoms occur. Possible non-drug causes of agranulocytosis are, in rare cases, bone marrow defects, e.g., myelodysplastic syndrome, sepsis triggered by bacterial or viral infections, splenomegaly, systemic lupus erythematosus and other autoimmune disorders or nutritional deficiencies, such as vitamin B_9_ (folic acid) or viramin B_12_ (cobalamin) deficiency (Andrès and Maloisel, 2008; Palmblad et al., 2014; Curtis, 2017).

Two groups of drugs can be distinguished as possible triggers of agranulocytosis. First, neutropenia up to agranulocytosis can occur particularly when cytotoxic agents are used in the therapy of cancer or with the high-dose use of cytotoxic immunosuppressants, e.g., azathioprine (Gregoriano et al., 2014) or methotrexate (Sun et al., 2017). The detrimental effect of these drugs is dose-dependent and can be anticipated due to the pharmacodynamics of these drugs. Neutropenia is the most important therapy-limiting factor in cancer chemotherapy and radiochemotherapy. Depending on the radiation dose, tumour radiotherapy can also cause DNA damage in bone marrow cells, leading to neutropenia of varying severity. Due to these known adverse drug reactions and problems, the blood count of such patients is closely monitored so that an early and rapid intervention can be made to any neutropenia or onset of agranulocytosis. As a rule, haematopoietic growth factors such as granulocyte colony stimulating factor (G-CSF, e.g., filgrastim, lenograstim, pegfilgrastim) or granulocyte monocyte colony stimulating factor (GM-CSF, e.g., sargramostin, molgramostin) are administered for treatment in such cases after interruption of the cytotoxic drug therapy. Dealing with the problems described is part of standard therapy management in the treatment of cancer or in the use of immunosuppressants with cytotoxic potential and will therefore not be discussed further in this review.

## Drugs as Triggers of Idiosyncratic Agranulocytosis

In contrast, there are a number of (non-cytotoxic) drugs with which cases of so-called “idiosyncratic” agranulocytosis are observed. Idiosyncratic drug side effects are also called type B reactions [B = “bizzare” (Naisbitt et al., 2000)] or hypersensitivity reactions and occur only rarely. Due to this rarity, it is difficult to reliably determine incidences of the occurrence of agranulocytosis with such drugs, even if they are known as potential triggers of agranulocytosis. In the case of an actual occurrence of drug-induced idiosyncratic agranulocytosis, this low incidence is nevertheless an additional risk factor, since neither the symptoms (see above) nor the relative rarity give reason to immediately consider this potentially life-threatening condition. To date, only very limited risk factors have been identified for idiosyncratic drug side effects that make their occurrence more likely and thus predictable. They are often linked to the presence of certain idiosyncrasies in the individual patient, i.e., the presence of certain rare genetic factors is usually required. Idiosyncratic drug side effects can occur in different forms. Most frequently, they manifest themselves in the form of skin reactions, such as urticaria, generalised exanthema, Steven-Johnson syndrome and the like. Furthermore, liver damage, e.g., with halothane, autoimmune reactions or haematological changes such as anaemia or agranulocytosis are observed (Uetrecht and Naisbitt, 2013). In the context of idiosycratic reactions, no clear dose dependence of the triggering substances can be observed. Nevertheless, these type B reactions are not completely dose-independent. Thus, the probability of triggering an idiosyncratic (type B) agranulocytosis increases when substances that can potentially trigger such a reaction are administered in higher doses. One can speculate that the risk is also increased in the sense of synergistic toxicity when several drugs with a potential risk of neutropenia or agranulocytosis are given at the same time. In addition, it has been observed that drugs given in doses of less than 10 mg/day cause virtually no idiosyncratic side effects [type B reactions] (Uetrecht, 1999).

Due to the low incidence of idiosyncratic reactions and the fact that in clinical trials prior to market approval (phase I to III of clinical drug development) narrow inclusion and exclusion criteria are often applied in the selection of subjects, it is not uncommon for these drug side effects to be noticed only after drug approval has been granted. Idiosyncratic agranulocytosis also has an average lethality of about 5%. Risk factors associated with a higher lethality of this form of agranulocytosis include an age of >65, a neutrophil count of <100 cells/µL and signs of sepsis, the last two characteristics often being considered “true” agranulocytosis in the eyes of haematologists (Andrès et al., 2006). As already mentioned, the fact that agranulocytosis is frequently recognised late also contributes to the considerable lethality of the disease. However, if an occurring idiosyncratic agranulocytosis is recognised early enough and the triggering drug is discontinued, the neutrophil count usually normalises after 1–2 weeks, provided no irreversible damage has occurred by then (Andersohn et al., 2007).

The overall annual incidence of drug-induced idiosyncratic agranulocytosis varies from study to study, possibly due to different evaluation methods or ethnic or population differences. In Europe an incidence between 1.6 and 9.2 cases per million population per year has been observed (Andersohn et al., 2007; Andrès and Maloisel, 2008); in the United States, an annual incidence ranging from 2,4 to 15,4 per million has been reported (Strom et al., 1992). Despite these low incidences, the risk of patients being affected by agranulocytosis due to drug treatment should not be underestimated, as there are a considerable number of drugs for which studies or case reports of the occurrence of agranulocytosis are available. Furthermore, it should be taken into account that severe neutropenia is usually detected in hospitalized patients, since the determination of the blood count is part of the usual routine diagnostics in the hospital. In contrast, a number of cases are likely to go undetected in the outpatient setting, e.g., when elderly outpatients die of or with agranulocytosis at home or because case registration is based on voluntary reporting. For these reasons, the incidence of agranulocytosis may be underestimated.

In patient populations that are treated with a specific agranulocytosis-risk drug the incidence rates range from a few single cases (Andrès et al., 2017) up to almost 1% in patients who were treated with clozapine (Alvir et al., 1993; Mijovic and MacCabe, 2020). Table 1 shows an overview of drugs that are associated with the triggering of ideosyncratic agranulocytosis (Andersohn et al., 2007; Andrès and Maloisel, 2008; Andres and Mourot-Cottet, 2017; Curtis, 2017; Stamer et al., 2017). It illustrates the variety of drugs that can be considered as triggers. However, this list is neither complete nor is it certain that each of the listed drugs is causally involved in the development of agranulocytosis (Andersohn et al., 2007; Ben Salem et al., 2008). This is due to the fact that idiosyncratic agranulocytosis is usually immunologically mediated. For this reason, it often occurs with a time lag, which fundamentally complicates the causal assignment to a specific exposure.

**TABLE 1:** Selection of drugs with high evidence of causing idiosyncratic agranulocytosis. Modified according to (Andersohn et al., 2007; Andrès and Mourot-Cottet, 2017).

Drug classes	Drugs
Analgesics/NSAID	Diclofenac, indomethacin, ibuprofen, metamizole, naproxen, acetaminophen, phenylbutazone, piroxicam
Antidepressants	Clomipramine, doxepin, fluoxetine, imipramine, maprotiline, mianserin
Anticonvulsants	Carbamazepine, phenytoin, lamotrigine
Anti-infectives	Abacavir, acyclovir, ampicillin, atovaquone, amoxicillin/clavulanic acid, cefotaxime, ceftriaxone, cefuroxime, cephalexin, clarithromycin, gentamicin, ganciclovir, imipenem/cilastatin, isoniazid, minocycline, nitrofurantoin, norfloxacin, oxacillin, penicillin G, piperacillin, rifampin, roxithromycin, terbinafine, trimethoprim/sulfamethoxazole, vancomycin, valganciclovir, zidovudine
Antineoplastic drugs	Flutamide, imatinib, rituximab
Antipsychotic drugs	Chlorpromazine, clozapine, levomepromazine, olanzapine, perazine, quetiapine, thioridazine, ziprasidone
Antirheumatic drugs	Auranofin, infliximab, penicillamine, sulfasalazine
Thyrostatic drugs	Carbimazole, thiamazole, Propylthiouracil
Cardiovascular drugs	(Acetyl-)Digoxin, amiodarone, bezafibrate, captopril, Doxazosin, methyldopa, procainamide, propranolol, ramipril, spironolactone
Gastrointestinal drugs	Cimetidine, famotidine, mesalamine, metoclopramide, omeprazole, pirenzepine, ranitidine
Antiplatelet drugs	Clopidogrel, dipyridamole, ticlopidine
Biologicals	Alemtuzumab, infliximab, rituximab, tozilizumab
Others	Acitretin, allopurinol, deferiprone, peginterferon alfa-2, prednisolone, promethazine, riluzole, levamisole

An international consensus meeting proposed the following criteria to assess the causal involvement of a drug in the development of agranulocytosis (Benichou and Solal Celigny, 1991): First, the occurrence of agranulocytosis during or up to 7 days after drug treatment with normalisation of the neutrophil count to >1,500/µL within 1 month after discontinuation of the drug. Second, the absence of the exclusion criteria “history of congenital or immune-mediated neutropenia”, “recent infection (especially viral)”, “prior chemotherapy or radiotherapy” or “therapy with biologicals, haematological disease”. Lastly, recurrence of neutropenia or agranulocytosis after repeated treatment with the drug (rechallenge). Although the latter criterion provides a high level of evidence, such an approach to systematic cause identification is not acceptable, as it may be associated with a considerable risk for the patient.

Understandably, a risk-benefit assessment for each drug with a potential risk of agranulocytosis must be carried out individually 1) with regard to the respective incidence of agranulocytosis, 2) the indication of the drug (risk of agranulocytosis versus the risk posed by the disease being treated) and 3) individual risk factors of the patient.

## Mechanisms in the Initiation of Idiosyncratic Agranulocytosis

The exact mechanisms leading to the development of idiosyncratic agranulocytosis have not yet been clarified. The possible mechanisms are discussed below and have been comprehensively addressed in a review by Johnston and Uetrecht in 2015 (Johnston and Uetrecht, 2015). The data available so far suggest that idiosyncratic agranulocytosis is based on the same mechanisms as other type B drug reactions.

## High Evidence for Immune-Mediated Mechanism

There is ample evidence that most of these reactions are immune-mediated. In contrast, there is little evidence for alternative hypotheses, e.g., toxic mechanisms (Sperner-Unterweger et al., 1993; Guest and Uetrecht, 1999; Williams et al., 2000; Johnston and Uetrecht, 2015), although it is possible that when an idiosyncratic response is triggered, both mechanisms may occur together or be jointly involved in triggering the drug side effect. For example, cell damage caused by toxic reactions can trigger signals that induce or stimulate immune responses (Séguin and Uetrecht, 2003; Weston and Uetrecht, 2014). The following frequently observed characteristics of agranulocytosis suggest an immunologically mediated mechanism in the majority of idiosyncratic reactions (Johnston and Uetrecht, 2015): First, a delayed onset of the reaction after initiation of the therapy [typically 1–6 months after first dose application depending on the drug; this period is presumably necessary to allow the clonal expansion of a pathologically relevant number of T lymphocytes with the necessary specificity and dominance, which subsequently trigger the clinical symptoms (Uetrecht and Naisbitt, 2013)]. In this regard, the delayed onset of the reaction after exposure may make it difficult to identify the triggering factor, especially if several factors are involved. Second, on the other hand, the rapid reappearance of the reaction after resumption of medication following interruption due to idiosyncratic reactions, a process termed “rechallenge”, which is due to the presence of corresponding memory T cells. Third, the appearance of eosinophilia and the formation of drug-dependent neutrophil antibodies or autoantibodies (Curtis, 2014; 2017), the appearance of which was first demonstrated 1952 (Moeschlin and Wagner, 1952). In these pioneering experiments, one of the investigators was able to induce agranulocytosis in himself by self-injection with serum from a patient with aminopyrine-induced agranulocytosis and concomitant aminopyrine treatment. Lastly, the association of idiosyncratic reactions with specific HLA gene variants (HLA = Human Leukocyte Antigen) (Fan et al., 2017).

The immune response may be triggered by the drug acting directly as an antigen, which is unlikely with small molecules but possible or even expected with biologicals. With small molecules, it is more likely that the drug or one of its metabolites acts as a hapten and binds covalently to a protein (Landsteiner and Jacobs, 1935). The resulting structural change in the affected protein (antigen formation) can lead to T-cell activation and trigger an immune response after uptake by antigen-presenting cells (APCs), cleavage into peptide fragments that takes place intracellularly here, and surface presentation of these (“foreign”) fragments via the major histocompatibility complex (MHC) to T-cells (Uetrecht, 2008; Johnston and Uetrecht, 2015). In order for the drug to act as a hapten, it is usually necessary that it itself or one of its metabolites has reactive, usually electrophilic structures that are able to react covalently with nucleophilic structures of proteins (NH_2_, SH, OH groups). Examples of such compounds with reactive structures are β-lactams (β-lactam ring; Parker et al., 1962) and 12-hydroxy-nevirapine sulphate as a reactive metabolite of the reverse transcriptase inhibitor nevirapine (Sharma et al., 2013). Furthermore, the metabolic epoxidation of aromatic rings, as is known, for example, from the gout agent benzbromarone (Wang et al., 2017), is a possibility for the formation of reactive metabolites that react covalently with proteins and can lead to the initiation of an immune response (in the case of benzbromarone with subsequent liver damage) (Wang et al., 2016). Reactive metabolites are most likely to be produced by CYP-mediated phase I botransformation reactions in the liver. This also explains why the liver is one of the organs in which idiosyncratic reactions can manifest (Uetrecht and Naisbitt, 2013). The formation of a hapten-protein adduct in relevant amounts does not mean that immune activation must occur. This seems to depend on the specific product formed, its ability to undergo structural changes and its reactivity towards other molecular structures, as well as on the presence of certain molecules interacting with the adduct formed. Especially the latter point might be a reason for the low incidence of idiosyncratic drug reactions and their association with certain HLA variants.

Other mechanisms of immune system activation are also discussed in connection with the pathogenesis of agranulocytosis. For example, the “danger hypothesis” propagated by Matzinger et al. assumes that cell damage triggered e.g. by reactive drug metabolites generates so-called “danger signals”, which in turn lead to the upregulation of co-stimulatory molecules (e.g., B7 and CD28), which represent the second activation signal necessary for T-cell activation (Anderson and Matzinger, 2000). Weston and Uetrecht found that the ability of drugs to activate inflammasomes and thus stimulate the formation and release of pro-inflammatory cytokines is associated with an increased risk of idiosyncratic side effects (Weston and Uetrecht, 2014). Other noxious agents, such as viral infections or surgical procedures, can also produce danger signals that increase the risk of idiosyncratic reactions, including agranulocytosis (Ellrodt et al., 1984; Levy, 1997). A further hypothesis (“The PI Concept” = pharmacological interaction with immune receptors) postulates that certain drugs can bind directly and non-covalently to T cell receptors and MHC molecules and thus activate T cells. This would be conceivable for drugs that do not form reactive metabolites (Pichler, 2002; 2003; 2005).

### Which Drug Properties are Important for the Initiation of Agranulocytosis?

Due to the variety of different drugs that can trigger agranulocytosis (Table 1) or other idiosyncratic reactions (Nakayama et al., 2009), it is difficult to derive rules by which chemical structures or general properties of drugs trigger an increased risk for such reactions. In order to identify chemical structures of drugs that are metabolised to reactive structures under *in vivo* conditions, one must know the metabolites of the corresponding drugs. In this context, however, it can be assumed that for many drugs the resulting metabolites are not all known or have not all been characterised. This is due to the fact that particularly reactive metabolites continue to react rapidly after their formation and can therefore only be detected - if at all - in very low concentrations. The rarity of idiosyncratic reactions also allows the assumption that certain (reactive) drug metabolites are possibly only formed in disease-relevant quantities in certain individuals, e.g. due to existing genetic polymorphisms. Nonetheless, in a study with 42 radiolabelled drugs of different risk classes for the formation of idiosyncratic reactions in three different hepatic test systems (human liver microsomes, hepatocytes *in vitro*, rat liver *in vivo*), Nakayama et al. found that two parameters of drugs correlate significantly with the empirically determined risk classes. First, the ability to form covalent bonds (due to existing reactive structural elements or due to the formation of reactive metabolites) and the daily dose (Nakayama et al., 2009).

### Why are Neutrophils Affected by Idiosyncratic Drug Effects?

Since - as already described - reactive metabolites continue to react rapidly, it is to be expected that the formation of covalent bonds to proteins is most likely to occur where reactive metabolites are formed. The form of any idiosyncratic reaction that develops, or which cells and tissues are affected, may therefore depend on the metabolic capabilities of the respective cells with respect to the triggering drug. In the case of idiosyncratic agranulocytosis, it is therefore likely that biotransformations occurring in neutrophils or their precursor cells play a major role in the pathogenesis of the disease. If, on the other hand, less reactive metabolites with higher stability, higher bioavailability and longer half-life are formed, it is also possible, that the site of formation of the metabolites and effector tissue are not identical. This may also be because proteins are expressed in other cells and tissues that are more reactive and thus also form covalent bonds with less reactive metabolites. If these metabolites can bind covalently to proteins in different tissues, it may thus also be possible that the same metabolic reaction leads to different idiosyncratic reactions (skin reaction, liver damage, lowering of the neutrophil count) in different individuals (Johnston and Uetrecht, 2015).

The most important biotransformation organ is the liver, in which oxidation processes catalysed by CYP enzymes in particular can lead to the formation of reactive drug metabolites, so that liver damage is also a possible form of idiosyncratic drug reactions (Uetrecht and Naisbitt, 2013). For many drugs that are metabolised in the liver, in the case of orally ingested drugs possibly already during the first liver passage, the hepatic biotransformation could be the starting point of idiosyncratic drug reactions in other cells and tissues due to the formation of reactive metabolites with a longer half-life. In neutrophils, the major oxidative biotransformation system is the peroxide-producing NADPH oxidase system together with myeloperoxidase, which uses the resulting hydrogen peroxide to oxidise chloride into hypochlorite. Neutrophils use hypochlorite to destroy pathogens, however, this also allows oxidation of drugs (Uetrecht, 1992b). In addition to neutrophils, sufficient amounts of myeloperoxidase are also found in myeloid progenitor cells, beginning with promyelocytes, as well as in monocytes and macrophages (Brown et al., 2001; Johnston and Uetrecht, 2015). Yet the oxidative potential of myeloperoxidase is not as pronounced as that of the CYP enzymes that are predominantly active in the liver. Therefore, mainly more easily oxidisable drugs are oxidised to reactive metabolites by this enzyme system. These include drugs with aromatic amino functions (e.g., procainamide, sulfonamides, aminosalicylic acid), thiourea derivatives (e.g., thiamazole, carbimazole, propylthiouracil), compounds that easily oxidise to quinones or quinonimines (e.g., carbamazepine, amodiaquine, phenothiazines) as well as other drugs such as metamizole and clozapine (Liu and Uetrecht, 1995; Ju and Uetrecht, 1998; Lai et al., 1999; Johnston and Uetrecht, 2015). Moreover, there appear to be other oxidative systems in these cells that may contribute to the formation of reactive metabolites. For example, although myeloperoxidase knock-out mice showed a reduced rate of covalent binding of the antimalarial drug amodiaquine to bone marrow cells, this was still clearly detectable (Lobach and Uetrecht, 2014). Even in patients who genetically had low myeloperoxidase activity, the tendency to develop clozapine-induced agranulocytosis was not reduced (Mosyagin et al., 2004). Nonetheless, the involvement of myeloperoxidase in these processes is supported by the fact that basophilic granulocytes, which have no peroxidase activity, remain unaffected in agranulocytosis (Besser et al., 2009). This is further supported by the fact that in the bone marrow of agranulocytosis patients, a deficiency of myeloid progenitor cells down to the promyelocyte stage is typically observed. From this stage onwards, all progenitor cells show appreciable myeloperoxidase activity (Uetrecht, 1992b).

## Drugs With a Significant Risk of Idiosyncratic Agranulocytosis

Of the numerous drugs for which idiosyncratic agranulocytosis has been observed and reported (Table 1), some are particularly known for their significant risk of agranulocytosis. There may be several reasons why such drugs remain on the market despite the potentially life-threatening effects of any agranulocytosis that may occur. First, Idiosyncratic agranulocytosis remains a rare adverse event even with the respective drug, so that the risk-benefit assessment, also taking into account the consequences of the disease to be treated, is in favour of the drug. Second, the use of such drugs may be justified by the fact that there are no or only limited therapeutic alternatives for certain indications (e.g., clozapine in the treatment of therapy-resistant schizophrenia or metamizole for therapy-resistant fever). Thus, the use of such drugs is also an indicator that there is a need for further developments in drug therapy for these indications. If lower-risk substances are developed, these “high risk” drugs usually lose clinical importance. For example, the platelet aggregation inhibitor ticlopidine, which is known for its agranulocytosis side effect, is now largely replaced by other antiplatelet drugs (clopidogrel, prasugrel, ticagrelor) that do not possess this side effect. Third, the use of these drugs can be acceptable, if the blood count and especially the neutrophil count are closely monitored as part of the drug safety management and especially at the beginning of the treatment. Any neutropenia or agranulocytosis that may develop can thus be detected at an early stage and appropriate countermeasures (discontinuation of the drug, treatment with granulocyte colony stimulating factor - G-CSF) can be initiated. Fourth, the risk of idiosyncratic agranulocytosis appears to be population-related, e.g., associated with certain genetic traits and HLA gene variants. Thus, use of drugs in populations with a low risk may therefore be considered acceptable.

In addition, after exceeding the typical treatment duration (depending on the drug) within which idiosyncratic agranulocytosis usually occurs (time to onset), the risk of such an event decreases significantly. Of course, it must be taken into account that agranulocytosis can also occur after this period. The “typical”“ time to onset of agranulocytosis after the start of therapy is shown for important drugs with a risk of agranulocytosis in Table 2 (Andersohn et al., 2007). After expiry of the typical “time to onset” (plus safety margin), the intervals of the therapy-accompanying monitoring activities (blood count checks) can generally be extended.

**TABLE 2:** Median treatment time until the occurrence of idiosyncratic agranulocytosis; adapted from (Andersohn et al., 2007).

Drugs	Median duration of treatment until onset of idiosyncratic agranulocytosis (duration in days)
Metamizole (dipyrone)	2
Rituximab	4 (median number of infusions until onset)
Cefapirine	19
Oxacillin	22
Nafcillin	22
Penicillin G	25
Aprindine	29
Captopril	32
Ranitidine	33
Quinidine	35
Mianserine	35
Propylthiouracil	36
Ticlopidine	39
Dapsone	39
Carbimazole	41
Terbinafine	42
Sulfalazine	42
Methimazole	42
Chlorpromazine	45
Amodiaquine	46
Procainamide	47
Carbamazepine	49
Clozapine	56
Levamisole	60

In the following, some important medicinal products with a relevant risk of idiosyncratic agranulocytosis will be discussed. They are also intended to illustrate, by way of example, the interrelationships and problems that are of significance for drugs with a higher risk of agranulocytosis.

### Metamizole (Synonyms: Novamine Sulfone, Dipyrone)

Metamizole is an analgesic used for moderate to severe pain, often as an alternative to NSAIDs or weak opioids. It also has an antipyretic effect and is used for fever that does not respond to other therapies. Due to its known risk of agranulocytosis, it is not approved in some countries, e.g., United States, Canada, United Kingdom, Sweden, Norway, Denmark. Nevertheless, the prescription figures in Germany increased continuously in the 2000s [from 87 million DDD in 2007 to over 200 million DDD in 2016 (Boeger et al., 2018)]. This may also be due to the fact that metamizole is available only on prescription and is paid for by the health insurance funds when prescribed. Since the use of metamizole does not require a dose adjustment in patients with renal insufficiency and because it is less harmful to the kidneys, it is often used - also “off label” - as an alternative to NSAIDs in older patients with renal insufficiency. However, it should be noted that metamizole is also associated with an increased risk of renal damage when used in high doses (3–10 g/day) and in critically ill patients (Glanzmann et al., 2016; Stueber et al., 2017). Due to the increasing prescriptions, the number of cases of agranulocytosis caused by metamizole has also increased in Germany in the past years. However, despite increasing prescriptions, the number of lethal agranulocytoses has remained almost constant in the years 1999–2009 (on average approximately 3–5 cases/year (Rotthauwe, 2011). Against this background, the increasing use of metamizole is justified, among other things, by the fact that, compared to the very rare cases of agranulocytosis, serious consequences of NSAID and opioid use are avoided to a much greater extent. In the evaluation of a German database on spontaneous reports of metamizole-induced agranulocytosis cases (Stammschulte et al., 2015), it was found that fatal courses were more frequently associated with methotrexate comedication. The analysis identified 12 patients with parallel methotrexate medication, 10 of whom died. On the one hand, this confirms that the simultaneous administration of cytotoxic, potentially neutropenia-inducing drugs increases the risk of (severe) agranulocytosis and at the same time shows how important it can generally be to analyse the circumstances accompanying therapy in order to avoid serious drug side effects.

The median time to onset of acute agranulocytosis after the start of therapy seems to be shorter with metamizole than with other drugs that cause agranulocytosis. It is reported to be around 2 days (Andersohn et al., 2007). Other studies found longer median time lags of 7 (Blaser et al., 2015) or even 13 days (Hedenmalm and Spigset, 2002). The latter study was conducted in Sweden after metamizole had been off the market for 20 years. For this reason, the study population consisted mainly of metamizole-naïve individuals. Therefore, it is reasonable to speculate that in populations with widespread use of metamizole, median time to onset of agranulocytosis is significantly lower, likely due to a greater proportion of carriers of metamizole-induced antibodies. This hypothesis would be in agreement with the study of Moeschlin and Wagner (Moeschlin and Wagner, 1952; see above). This could also lead to the conclusion that the extremely short median onset times of 2 days (Andersohn et al., 2007) may actually be rechallenge times and that then the shorter onset times in the treatment with metamizole should be taken into account when monitoring the blood count during therapy. A dependence of metamizole-induced agranulocytosis on certain HLA gene variants cannot be ruled out, but there is no clear evidence for this to date (Shah, 2019; Cismaru et al., 2020). Estimates of the incidence of agranulocytosis with metamizole treatment vary widely regionally and in the literature (from, for example, 1 case per 143,000 therapy cycles over 2 weeks in a Berlin study (Huber et al., 2015) to 1 case per 1,439 treatments in Sweden (Hedenmalm and Spigset, 2002). These differences may be due to genetic variability in the regional populations studied. Another cause may be different methodologies of data collection. Spontaneous reports are often used to estimate the incidence of rare outcomes (as in the case of agranulocytosis), which are voluntary, so milder cases may be reported less frequently and certain surveys may be associated with a high rate of unreported cases.

### Clozapine

Clozapine is one of the drugs with the highest incidence of agranulocytosis (up to almost 1% of users in a United States study (Alvir et al., 1993; Mijovic and MacCabe, 2020). The level of incidence appears to vary regionally. For example, a study in China found a much lower incidence of 0.21% (Tang et al., 2008). Clozapine belongs to the class of so-called atypical antipsychotics (Second Generation Antipsychotic Drugs), which have the advantage over other antipsychotics that they also improve negative symptoms (e.g., listlessness, social withdrawal, depression) in psychotic disorders and schizophrenia and cause significantly fewer extrapyramidal motor disorders (EPS), in particular fewer tardive dyskinesias as a side effect. Within this drug class, clozapine is characterised by the lowest rate of EPS (EPS rate <5%) with a strong antipsychotic effect. It is mainly used when other antipsychotics fail or when strong EPS occur during the use of conventional antipsychotics (SIGN, 2013; NICE, 2014; Samara et al., 2016; Siskind et al., 2016), as well as in Parkinson’s patients who experience hallucinations due to therapy with dopamine agonists (Seppi et al., 2011; DGN, 2016).

The occurrence of clozapine-induced agranulocytosis is associated with older age and the presence of certain HLA gene variants (e.g. HLA-B38, DRB1*0402, DRB4*0101, DQB1*0302, HLA-DQB1, HLA-B (Yunis et al., 1995; Legge and Walters, 2019), which could explain regional incidence differences. A reactive and short-lived nitrenium ion is assumed to be the trigger, which is formed by the action of myeloperoxidase on the clozapine metabolite N-desmethyl clozapine (Uetrecht, 1992a; Gerson et al., 1994). Binding of the nitrenium ion to neutrophil granulocyte structures promotes their apoptosis, which may be modified by genomic factors (Williams et al., 1997; Williams et al., 2000). Another assumption is that the binding of the nitrenium ion leads to changes in the granulocyte membrane and the formation of new antigens that trigger an immune response (Uetrecht et al., 1997; Williams et al., 2000). In almost all patients taking clozapine, covalent binding to neutrophils can be detected, although ultimately only about 1% develop agranulocytosis (Gardner et al., 1998). The median time to onset of idiosyncratic agranulocytosis is relatively long with clozapine [about 56 days (Andersohn et al., 2007)]. More than 50% of clozapine patients show an increase in inflammatory cytokines, a rise in temperature and an increased neutrophil count in the early phase of treatment (Pollmächer et al., 2000), which, however, does not necessarily indicate an emerging agranulocytosis, the incidence of which (see above) is significantly lower.

Due to the relatively high incidence of agranulocytosis, the prescription of clozapine in Germany is associated with special requirements: Written information for the patient, weekly blood count checks until the 18th week of therapy, then monthly until 4 weeks after discontinuation. Similar rules apply in many other countries (Mijovic and MacCabe, 2020). The discontinuation criteria for clozapine monitoring vary. In the United States, since 2015, the Food and Drug Administration (FDA) has specified the absolute neutrophil count (ANC) alone as the decisive discontinuation parameter with a less restrictive, lower threshold (Table 3) (Mijovic and MacCabe, 2020; FDA, 2015). In the United Kingdom, a traffic light system developed by the National Institute for Health and Care Excellence (NICE) is used, which also assesses the total white blood cell (WBC) count. Both systems also take into account people with benign ethnic neutropenia (BEN, Table 3) (Mijovic and MacCabe, 2020).

**TABLE 3:** Monitoring criteria for clozapine therapy in the United Kingdom and the United States; modified according to (Mijovic and MacCabe, 2020).

Country	Level	WBC (Leukocytes/µL)	ANC (Neutrophils/µL)	ANC in BEN (Neutrophils/µL)	Recommendation
United Kingdom (NICE)	Green	>3,500	>2000	>1,500	Continuation of therapy
	Amber	3,000–3,500	1,500–2000	1,000–1,500	Continuation of therapy at 2x weekly monitoring
	Red	<3,000	<1,500	<1,000	Discontinuation of therapy
United States (FDA)	—	—	<1,000	<500	Discontinuation of therapy

Studies to date have not demonstrated a higher risk of agranulocytosis when the less restrictive FDA criteria are applied (Sultan et al., 2017). Due to the unique efficacy of clozapine, which has not yet been achieved by any other lower-risk antipsychotic drug, there are repeated attempts to restart therapy (rechallenge) after discontinuation, despite regulatory requirements to the contrary (“off label”). A study on this problem showed that of 53 rechallenge patients, 20 (38%) experienced critical neutropenia again, of which 9 patients experienced agranulocytosis (Dunk et al., 2006). Thus, at the same time, rechallenge was successful in 62% of patients. In a study of 25 rechallenge patients co-medicated with lithium, only one patient (4%) experienced a recurrence of critical neutropenia, suggesting a lithium protective effect with clozapine rechallenge (Kanaan and Kerwin, 2006). In another study of patients whose ANC had not fallen below 500/µL when therapy was discontinued, clozapine rechallenge was successful in 79% of cases (Meyer et al., 2015). In contrast, a systematic review showed that in 80% of patients who experienced agranulocytosis at baseline (ANC< 500/µL), clozapine rechallenge failed (Manu et al., 2012). The results show that in patients with severe or refractory schizophrenia, a rechallenge is an option when the first attempt at therapy was discontinued due to leukopenia or mild neutropenia, but not due to agranulocytosis. Such a rechallenge should be closely monitored haematologically and co-medication with lithium or granulocyte colony-stimulating factor (G-CSF) may support therapy. Concomitant medications with neutropenia potential should be discontinued or replaced with lower-risk alternatives (Mijovic and MacCabe, 2020).

Other antipsychotic and psychotropic drugs are also known to trigger idiosyncratic agranulocytosis (Table 1), but with a much lower incidence. This may also be a dosage effect. For example, olanzapine, which is structurally related and has a similar pharmacological effect, is usually used in doses 20–40 times lower than clozapine.

### Thyrostatic Drugs [Propylthiouracil, Thiamazole (Methimazole), Carbimazole]

The named thyreostatics are inhibitors of thyroid peroxidase. This blocks the conversion of iodide to iodine, the incorporation of iodine into tyrosine residues and the conversion of iodinated tyrosines into iodine-thyronines (iodisation inhibitors). Likewise, the activation of thyroxine (T4) to triiodothyronine (T3) is reduced. The totality of these processes leads to a decrease in thyroid hormone levels. In 0.3–0.7% of treated patients, idiosyncratic agranulocytosis develops (Pick and Nystrom, 2014). This occurs preferentially in the second-third month after the start of treatment, but can also occur later. The fact that thyrostatic drugs trigger this side effect may be due to the similarity between thyroid peroxidase and the myeloperoxidase of neutrophils (Huang et al., 2007). A possible interaction of thyrostatic drugs with myeloperoxidase may therefore also lead to the formation of reactive metabolites in neutrophils and thus to the formation of modified proteins that act as antigens and contribute to the initiation of agranulocytosis. In the case of propylthiouracil, the formation of circulating so-called anti-neutrophil cytoplasmatic antibodies (ANCA) was observed. These react with antigens found in the granules of neutrophils when they migrate to the cell surface. This can lead to the induction of apoptosis (Fibbe et al., 1986; Gilligan et al., 1996). Among white Europeans, carriers of the HLA-B*27:05 gene or certain single nucleotide polymorphisms (SNPs) on chromosome 6 have an increased risk of thyrostatic-induced agranulocytosis (Hallberg et al., 2016).

Of the three thyreostatics mentioned, the incidence of agranulocytosis is highest with propylthiouracil use (approx. 0.7% and thus approx. 2.7-fold higher than with carbimazole (Hallberg et al., 2016). A dosage effect is also conceivable in this case, as propylthiouracil is dosed approximately 10-fold higher than carbimazole and thiamazole. However, since the latter substances are prescribed more frequently, most thyrostatic-induced agranulocytoses are caused by these substances, in France, for example, 87% (Andrès et al., 2016).

### Levamisole

Levamisole (the levo enantiomer of tetramisol) is used as an anthelmintic in veterinary medicine. It acts as an acetylcholine agonist on the nicotinic receptors of the ganglia and the motor end plate of the parasites, thus paralysing their locomotor system. In addition, it also has immunomodulatory and immunostimulatory properties. It has therefore been used as an antiarthritic agent and in the treatment of colon carcinoma in combination with fluorouracil (Amery and Bruynseels, 1992). In Germany and many other countries, levamisole is not approved for use in humans due to its side effects, e.g., agranulocytosis (Graber et al., 1976; Leca et al., 1976; Rosenthal et al., 1976; Ruuskanen et al., 1976; Sany et al., 1976), vasculitis and skin necrosis (Macfarlane and Bacon, 1978; Scheinberg et al., 1978) as well as pulmonary hypertension due to its biotransformation to aminorex (Fishman, 1999). However, levamisole is still important in human medicine as an agranulocytosis-inducing substance, as it has been used as an extender for illegally trafficked cocaine since the beginning of the 2,000s (Larocque and Hoffman, 2012). The proportion of levamisole varies from about 1% to well over 10%. The reason for its use as an extender is seen in the fact that levamisole also induces states of arousal (metabolite aminorex) and thus “boosts” the effect of cocaine. The user is thus led to believe that the product is particularly “pure”, despite the fact that the substance has been blended (Erowid Crew, 2009; Larocque and Hoffman, 2012). In 2009, already more than 50% of the cocaine samples in Great Britain and the Netherlands were spiked with levamisole (Erowid Crew, 2009). In 2016, a warning was issued in Switzerland against the consumption of cocaine containing levamisole, which at that time was found in approxiately 70% of the samples with a mean proportion of 13.2% (Tox Info Suisse, 2016). The intake of Levamisole in this way without the user`s knowledge is highly problematic and may have serious consequences (Kinzie, 2009; Zhu et al., 2009). In people with neutropenia who otherwise hardly take any drugs, cocaine abuse should therefore also be considered.

In clinical studies, agranulocytosis was observed in 0.8–5.0% of the respective study participants when taking 50–200 mg levamisole/day (US Department of Justice, 2019). Levamisole-induced agranulocytosis is characterised by a characteristically long onset delay (approximately 60 days; Table 2). HLA-B27 carriers probably have an increased risk of agranulocytosis when taking levamisole (Schmidt and Mueller-Eckhardt, 1977; Mielants and Veys, 1978; Veys et al., 1978). However, a number of cases of leukopenia and agranulocytosis were observed in patients without HLA-B27 suggesting a multifactorial mechanism (Vogel et al., 1980). A levamisol-induced agranulocytosis typically reversed upon discontinuation of the substance (Mielants and Veys, 1978).

### Anti-Infectives

Anti-infectives represent the class of drugs with the highest incidence of agranulocytosis outside oncological treatments, especially β-lactams and cotrimoxazole (combination of sulfamethoxazole and trimethoprim), as well as some antiviral compounds (van Staa et al., 2003; Andrès et al., 2017). Since in most cases these drugs are only used for a short time, substances with a short onset delay and/or reactive structure (β-lactams) are more likely to be the trigger. However, it is also possible that cases of agranulocytosis occur due to repeated use of the same or a similar substance whose initial use has already induced immunological reactions. Anti-infectives are more difficult to evaluate in terms of their role as causal triggers, as especially in bacterially induced sepsis or viral infections, the disease itself can lead to neutropenia or agranulocytosis. The majority of cases occurring under anti-infectives were observed in hospitalised patients (Andrès et al., 2017).

The fact that the combination of sulfamethoxazole and trimethoprim (an inhibitor of bacterial dihydrofolic acid reductase = DHFR) leads relatively more frequently to agranulocytosis could indicate that an influence on folic acid metabolism makes the highly proliferating cells of the haematopoietic system more sensitive to substances that trigger neutropenia. A similar effect has already been reported with the combination of methotrexate (also a DHFR inhibitor) with metamizole [see above (Stammschulte et al., 2015)]. However, human DHFR is inhibited by trimethoprim to a much lesser extent than bacterial DHFR (Brogden et al., 1982).

### Biologicals (Biotherapeutics)

Among the biologicals that are not used in the context of cancer treatment, a number of substances have been identified as potential triggers of idiosyncratic neutropenias and agranulocytoses. Relevant are drug classes and preparations that are mainly used in auto-inflammatory and autoimmune diseases: Tumour necrosis factor-α (TNF-α) inhibitors (adalimumab, infliximab, etanercept), CD20 antibodies (rituximab), CD52 antibodies (alemtuzumab), interleukin-6 (IL-6) antibodies (tocilizumab), IL-1 antagonists (anakinra, canakinumab) and B-cell activating factor inhibitors (belimumab) (Andrès et al., 2019). When assessing these biotherapeutics, however, it must be taken into account that due to the mechanisms of action of the drugs, neutropenia with neutrophil counts (ANC) of 1,500–500/µL as a side effect is not uncommon and occurs in approximately 10–15% of treated patients across all regimens. For this reason, such therapy can still be continued with ANC of 1,500–1,000/µL under close monitoring (Andrès et al., 2019). With ANC values below 1,000/µL, therapy should be temporarily suspended. After normalisation (ANC >1,500/µL), an attempt can be made to continue therapy. Agranulocytosis (ANC <500/µL) is reported much less frequently with such therapies (approximately 1–2%), although it has been observed particularly with rituximab and alemtuzumab. In such cases, therapy should be discontinued immediately and switched to an alternative treatment (Andrès et al., 2019). A characteristic feature of biotherapies is sometimes a so-called “late onset neutropenia”, which is characterised by low neutrophil counts (<500/µL) and a marked late onset from 3 weeks after administration to 3 months after the end of therapy (Salmon et al., 2015; Yiannopoulou et al., 2018; Andrès et al., 2019). Unlike small chemical molecules, induction of neutropenia by chemically reactive metabolites is not expected with biologicals. The induction of immunological reactions (Johnston and Uetrecht, 2015) and the influencing of haematopoietic processes are likely triggers.

Other drugs that are frequently mentioned in the literature in connection with the triggering of agranulocytoses are the antiepileptic drugs carbamazepine and phenytoin, the iron chelator deferiprone and the sulphasalazine used in chronic inflammatory bowel diseases and polyarthritis (see also Table 1).

## Prevention and Treatment of Idiosyncratic Agranulocytosis

Preventive measures should be taken, especially with drugs known for an increased incidence of idiosyncratic agranulocytosis (see above). Before prescribing, it should already be checked whether other drugs with inherent neutropenia potential are used in the patient. This also applies to prescriptions that are added later. Due to the large number of medicinal products with agranulocytosis potential (Table 1), a medication plan should always be prepared, which also chronologically documents the prescription times of the drugs used. This can help to identify the triggering drug(s), especially in the case of polymedication regimens. Patients or relatives and caregivers should be adequately informed in written form about the risk and symptoms of agranulocytosis and encouraged to contact the doctor if they occur. At the start of therapy, the blood count should be closely monitored over a sufficiently long period of time (median onset time plus safety margin). The most important monitoring parameter is the neutrophil count (ANC). When considering the threshold at which treatment should be discontinued, it makes sense to follow the guidelines for clozapine treatment (see above).

If critical neutropenia develops, all drugs that could be a trigger should be discontinued. If agranulocytosis is manifest (ANC <500/µL), the patient should be hospitalised and isolated, if not already done. Care in a haematology department with strict clinical and microbiological monitoring is recommended (Mijovic and MacCabe, 2020). The administration of granulocyte colony-stimulating factor (G-CSF) is generally recommended [e.g., Filgrastim 300 μg/d, Lenograstim 263 µg/] (Andrès and Mourot-Cottet, 2017; Mijovic and MacCabe, 2020). Nevertheless, there is a lack of high-quality studies that prove a shortening of the duration of neutropenia in idiosyncratic agranulocytosis by G-CSF. Previous studies with sometimes small numbers of subjects and different doses yielded contradictory results (Fukata et al., 1993; Fukata et al., 1999; Andersohn et al., 2007). More recent studies indicate an efficacy of G-CSF (Andrès et al., 2010; Lally et al., 2017), which can lead to a shortening of agranulocytosis by 4–5 days and an improvement in prognosis (Lally et al., 2017). If fever occurs or sepsis develops, antibiotics should be administered immediately according to the guidelines for the treatment of febrile neutropenia. Intravenous administration of broad-spectrum antibiotics is usually used.

All cases of drug-induced neutropenia or agranulocytosis should be consistently reported to the appropriate authorities or pharmacovigilance centres. Given the rarity of these events, these reports are important contributions for gaining further knowledge.

## Conclusion

Drug-induced agranulocytosis is one of the most serious adverse reactions and can occur as a problem in therapy with a wide variety of drug classes. In this regard, numerous drugs are able to trigger a rare idiosyncratic agranulocytosis. Initiators of idiosyncratic reactions are thought to be chemically reactive drugs or reactive metabolites that react with proteins and can subsequently cause an immune response. Cells or organs that exhibit specific metabolic activity are therefore frequently affected. Toxic reactions and direct immunological interactions, e.g., with biologicals, have also been associated with idiosyncratic reactions. The manifestation of agranulocytosis is typically delayed and potentially life-threatening. When prescribing drugs with a known increased risk of agranulocytosis, patients should be informed about possible symptoms. At the beginning of the therapy, the entire medication should be reviewed and the blood count closely monitored. If agranulocytosis occurs, all potentially triggering drugs should be discontinued immediately. Hospitalisation under haematological care is recommended. The use of G-CSF should be considered. In severe agranulocytosis, e.g., with sepsis, further measures should be taken according to guidelines. Agranulocytosis and neutropenia, which occur as adverse drug reactions outside of (cancer) chemotherapy, must be consistently reported to the responsible pharmacovigilance centres in order to improve the often incomplete or insufficient data on the agranulocytosis potential of drugs and thus ultimately also drug therapy safety management.
